# Comprehensive Review on Hair Loss and Restorative Techniques: Advances in Diagnostic, Artistry, and Surgical Innovation

**DOI:** 10.7759/cureus.82991

**Published:** 2025-04-25

**Authors:** Luis A Mendoza, Genaro G Ocampo, Yozahandy A Abarca-Pineda, Mubashir Ahmad Khan, Yasmin Ahmadi, Najaee Brown, Denyse Deowan, Zahra Nazir

**Affiliations:** 1 Medicine and Surgery, Universidad Cristóbal Colón, Veracruz, MEX; 2 Medicine and Surgery, Westhill University, Mexico City, MEX; 3 Internal Medicine, Hospital Médica Sur, Mexico City, MEX; 4 School of Medicine and Health Sciences, Tecnológico de Monterrey, Mexico City, MEX; 5 Medicine and Surgery, Baqai Medical University, Karachi, PAK; 6 Internal Medicine, Royal Shrewsbury Hospital, Shrewsbury, GBR; 7 Medicine and Surgery, University Hospital of the West Indies Mona Campus, Kingston, JAM; 8 Medicine and Surgery, San Fernando Teaching Hospital, San Fernando, TTO; 9 Internal Medicine, Combined Military Hospital, Quetta, Quetta, PAK

**Keywords:** 5-alpha reductase inhibitors, androgenic alopecia, minoxidil, prp vs minoxidil, telogen effluvium

## Abstract

Hair loss, or alopecia, is a complex disorder that impacts individuals worldwide, frequently resulting in significant psychological and social consequences. This review analyzes the multifactorial etiology, recent diagnostic innovations, and emerging treatment alternatives for hair loss management. Alopecia is classified into the cicatricial (scarring) and non-cicatricial (non-scarring) forms, each having a unique underlying pathogenesis, ranging from autoimmune dysregulation, androgenetic mechanisms, and environmental factors. Recent advancements in diagnostics, such as artificial intelligence (AI)-enhanced imaging and biomarker analysis, have improved precision and individualization of treatment. Novel therapies, such as low-dose oral minoxidil (LDOM), topical 5-alpha reductase inhibitors, and Janus kinase inhibitors (JAKi), offer a range of promising options for hair loss management. Non-invasive therapies, such as low-level laser therapy (LLLT) and platelet-rich plasma (PRP) injections, have demonstrated synergistic benefits with existing treatments. Surgical advancements, especially AI-assisted robotic follicular unit extraction (FUE), enhance precision and outcomes. Emerging trends in regenerative medicine, especially stem-cell-based therapies and AI integration, are influencing the future of customized hair restoration. This review serves as a comprehensive guide, highlighting the use of innovative technologies and therapies in enhancing the accuracy and customization of hair loss treatment.

## Introduction and background

Hair loss, or alopecia, is a prevalent condition affecting individuals across all ages, genders, and backgrounds, underscoring its universal impact on personal and societal well-being. Though often perceived as a cosmetic issue, its profound effects on self-esteem and mental health require treatment at times, given the type of hair loss and its severity [1]. Diagnostics, medical, and surgical innovations have transformed hair restoration, providing results that integrate functionality and aesthetics [2].

Alopecia is classified into two primary categories: cicatricial (scarring) and non-cicatricial (non-scarring). Cicatricial alopecia results from permanent follicular destruction, often due to inflammatory or traumatic factors, while non-cicatricial alopecia (non-scarring) preserves follicular openings and remains potentially reversible [3]. Diagnostic advancements, including trichoscopy and video dermatoscopy, have improved the accuracy and early detection of various forms of hair loss, enabling timely interventions [4].

Recent advancements in hair restoration have been transformative, particularly in refining surgical and non-surgical techniques. Innovations such as robotic hair restoration systems, which enhance precision in follicular unit extraction (FUE), have significantly improved outcomes by reducing graft damage and increasing procedural efficiency [5]. Equally transformative are innovations in artistry, which utilize a profound comprehension of hairline aesthetics to produce imperceptible, natural results [6].

Adjuvant therapies, including platelet-rich plasma (PRP) and low-level laser therapy (LLLT), are now widely recognized for their role in enhancing hair density and quality when combined with surgical procedures [2]. Stem cell-based therapies, aimed at addressing the limitations of donor hair availability, are an exciting frontier, with emerging evidence supporting their potential to revolutionize the field [7]. Mesenchymal stem cells, in particular those derived from adipose tissues and stem cells, have immunomodulatory properties that result in suppressing autoreactive T cells. This eventually leads to reduced local inflammation in the scalp.

These technological and procedural advancements underscore the evolution of hair restoration into a sophisticated medical discipline that effectively combines functionality with artistry to achieve superior aesthetic and clinical results. Our paper aims to shed light on using innovative technologies and therapies to enhance the accuracy and customization of hair loss treatment.

For this narrative review, a literature search was done using PubMed, Scopus, and Google Scholar. The included studies were in English and published in the last 10 years. The keywords used were alopecia, hair loss treatment, PRP, and trichology. Relevant peer-reviewed articles, reviews, and clinical studies were included based on relevance to the topic. No formal risk-of-bias assessment or quantitative synthesis was performed, as this review is descriptive in nature.

## Review

Hair follicle anatomy and growth cycle

Hair serves critical functions such as protection, heat insulation, sensory perception, and social interaction [8]. Hair follicles (HFs) are skin appendages derived from epidermal invagination during development, consisting of three segments: the infundibulum, isthmus, and inferior segment, the last one forming the hair bulb. The dermal papilla (DP) is at the bulb's base, which supplies blood and acts as a signaling center regulating hair growth through interactions with stem cells and WNT signaling. The DP is surrounded by a dermal sheath containing progenitor cells with wound-healing properties. Matrix cells within the bulb proliferate to extend the hair follicle and hair. Within the hair bulb, surrounding the dermal papilla, are other unspecialized epithelial cells known as matrix cells, which act as germ cells that grow the hair follicle and hair [9].

The HF growth cycle alternates among three phases: rapid growth (anagen), regression (catagen), and rest (telogen) [10]. Mature HFs are complex structures of concentric epithelial layers and dermal papilla cells (DPCs) originating from dermal stem cells (DSCs) during embryonic development. DSCs, which can stimulate epithelial hair follicle stem cells (HFSCs), are widely studied as key controllers of the HF growth cycle [3,11]. Despite protective mechanisms, hair abnormalities or loss (alopecia) commonly occur in males and females of all ages [3,9].

Alopecia is categorized into the scarring (cicatricial, CA) and nonscarring (noncicatricial, non-CA) types, which can be diffuse or patchy, congenital or acquired. Scarring alopecia involves permanent hair loss due to follicle destruction, while non-scarring alopecia preserves follicular openings and is potentially reversible [3,11,12]. Table 1 categorizes the subtypes of CA.

**Table 1:** Scarring alopecia Table created by the authors using data from [13].

Subtype	Mechanism	Clinical Features	Examples
Primary CA	- Direct inflammatory damage to the hair follicle - Inflammatory infiltrates (lymphocytic, neutrophilic, or mixed)	- Follicular destruction - Loss of follicular openings - Scalp burning, itching, tenderness	Frontal fibrosing alopecia, central centrifugal cicatricial alopecia, lichen planopilaris
Secondary CA	- Chronic inflammation secondary to external factors (e.g., trauma, infection, toxins)	- Follicular atrophy - Permanent scarring - Associated with external insults	Traction alopecia, chemotherapy-induced alopecia

Primary CA results from direct follicular inflammation, while secondary CA arises from external factors like traction or chemotherapy. Table 2 describes the different types of scarring alopecia.

**Table 2:** Scarring alopecia and its types Table created by the authors using data from: [14,15]

Type	Significant features	Hair Pull Test	Trichoscopy	Treatment and comments
ACNE KELOIDALIS NUCHAE (AKN)	Papules, pustules, and keloid masses occurring in the occiput and nuchal region.	Negative	Early stages: Erythematous papules and pustules, perifollicular scales, and hemorrhagic and honey-colored crusts. Advanced stages: Perifollicular white halos, black dots, broken hair shafts, dilated follicular ostia, and keratin or hair tufts. Keloid-like lesions: Milky-red areas with no follicular ostia	Overall, the treatment of AKN is difficult and unsatisfactory. Patient education, avoiding close shaving and frequent haircuts, topical antimicrobials, mild keratolytic agents, or topical steroids with or without a retinoid.
CENTRAL CENTRIFUGAL CICATRICIAL ALOPECIA (CCCA)	Occurs centrally and spreads peripherally and symmetrically.	Positive	Asterisk-like brown blotches, broken hairs, and dark peripilar halos.	Topical minoxidil, topical fluocinonide and ketoconazole shampoo. Intralesional triamcinolone injections (alone, with topicals, or with topicals and oral medication).
DISCOID LUPUS ERYTHEMATOSUS	Erythematous alopecic patch with follicular hyperkeratosis and areas of hyper- and hypopigmentation occurring on the scalp. Patients can also have lesions located on the face and ears.	Positive if concurrent with other kinds of alopecia (TE, AE, AA, etc.).	Thick arborizing vessels, follicular keratotic plugs, follicular red dots, peripilar scale, and peripilar erythema. Blue-gray speckled dots and blue-white veil are two features observed in patients with Fitzpatrick skin types IV-VI.	First-line therapy includes: Topical clobetasol under occlusion 4-5 days/week + Topical minoxidil.
DISSECTING CELLULITIS	Alopecia occurring most commonly on the vertex and occiput that results from multiple firm to fluctuant inflammatory nodules, abscesses, and plaques on the scalp, with an overlying patch of alopecia.	Positive, revealing broken hairs at different lengths.	Yellow dots: A 3D structure over dystrophic hair shafts is a characteristic finding. Early stage of dissecting cellulitis. Yellow structureless areas: These areas are "lakes of pus" that are often found around hair follicles. Black dots: Often found in active lesions. Pinpoint-like vessels: These vessels have a whitish halo and can be observed. Polytrichia: This is the emergence of five or more shafts per follicular unit. Skin clefts with emergent hairs: These are skin folds that contain shafts. White dots and amorphous white areas: These represent empty follicular units replaced by fibrosis. Blue-gray dots: These dots have a histopathological correspondence to pigmentary incontinence.	Comprises a variety of medications and other therapies: antibiotics (erythromycin), steroids (prednisolone), retinoids (oral or topical), and surgical excision, split-thickness skin grafting, or laser hair removal are considered for unresponsive cases.
FRONTAL FIBROSING ALOPECIA	Alopecic band occurring along the fronto-temporal region and often includes eyebrows. Can present with a pseudo-fringe sign, which is when some hair is retained along the original hairline.	Negative.	Loss of follicular openings (on the frontotemporal hairline), perifollicular erythema, follicular hyperkeratosis, absence of vellus hairs and solitary terminal hairs (both helpful clinical clues for diagnosis). Other findings are: yellow dots, perifollicular blue-gray dots, etc.	Has no cure. Can help slow progression: antibiotics, intralesional corticosteroids or as cream, finasteride or dutasteride, hydroxychloroquine, minoxidil.
LICHEN PLANOPILARIS (LPP)	Can occur in patchy, diffuse, or patterned form. Patchy: Alopecia occurs in small patches and merges to form larger alopecic patches. Diffuse: Alopecia generally starts at the crown. Patterned: Blend of androgenic alopecia and diffuse LPP.	Positive, used to measure the activity of lichen planopilaris (lichen planopilaris activity index, LPPAI).	Inflammation is mainly folliculocentric, erythema and scaling will have a perifollicular pattern of distribution. Scales form tubular structures around shafts, called casts. Small tufts containing 2-4 hair shafts surrounded by scaling are very suggestive of the diagnosis. Pili torti are commonly observed, but is also seen in all forms of primary cicatricial alopecias.	Comprises a variety of therapies: topical treatments (cortisone lotions, retinoids, calcineurin inhibitors), oral treatments (hydroxychloroquine, tetracyclines, immunosuppressants), intralesional corticosteroids, low-level laser therapy, etc, antimalarial drugs.

Non-CA includes androgenetic alopecia (AGA) and alopecia areata (AA), both common and extensively studied [3,8,10]. Table 3 summarizes the different types of non-scarring alopecia in detail.

**Table 3:** Non-scarring alopecia and its types Table created by the authors using data from: [13]

Subtype	Mechanism	Clinical Features	Examples
Patchy	- Immune-mediated or localized disruption of follicular function	- Localized hair loss - Preserved follicular openings - Potential for regrowth	Tinea capitis, trichotillomania
Diffuse	- Systemic or environmental factors affecting the hair growth cycle	- Generalized thinning or shedding - No scarring - Potentially reversible	Telogen effluvium (TE), anagen effluvium

Androgenetic alopecia (AGA)

AGA is the most frequent cause of hair loss as a consequence of hormonal factors and genetic predisposition, affecting 30-50% of men (male-pattern hair loss (MPHL)) and approximately 30% of middle-aged women (female-pattern hair loss (FPHL)). The mechanisms of AGA are multiple, interlinked, and common to both MPHL and FPHL. Among them is the hypothesis of oxidative stress (OS) resulting from increased expression of pro-inflammatory cytokines attributable to chronic perifollicular microinflammation. In the genetically predisposed (polygenic dominant with variable penetrance), OS works in tandem with high androgen levels and various environmental factors to interrupt the corticotropin-releasing hormone pathway and cortisol levels that lead to AGA. Moreover, gene variants play a critical role in the etiology of AGA by increasing the activity of 5a-reductase or the sensitivity of androgen receptors [14,15]. AGA is characterized by hair follicle (HF) miniaturization caused by dysregulation of the hair growth cycle, primarily due to the action of dihydrotestosterone (DHT). DHT binds to androgen receptors in the dermal papilla cells (DPCs) of susceptible hair follicles, initiating a cascade of molecular signals altering the expression of key growth-regulating genes, such as TGF-β, DKK-1, and IGF-1, which in turn lead to shortening of the anagen phase, follicular miniaturization, and eventual hair thinning. Histological analysis reveals that lymphocytes and mast cells accumulate around the miniaturized HFs and bulge area, accompanied by a decrease in proliferating progenitor cells despite an intact quantity of HFSCs. The presence of perifollicular infiltration and the involvement of inflammatory genes encoding caspase-7 and tumor necrosis factor (TNF) support the inflammatory hypothesis in AGA. This leads to the release of pro-inflammatory cytokines, such as interleukin (IL)-1, IL-6, and TNF-α, contributing to chronic microinflammation. Instead, ROS are continuously produced and degraded, but when oxidative stress (OS) overwhelms the follicle’s antioxidant defenses, damage occurs. This exceeds the follicular antioxidant defense capacity, resulting in premature and dysfunctional hDPCs. If left untreated and with exacerbation, AGA becomes irreversible [16].

Alopecia areata (AA)

AA is an autoimmune, non-scarring alopecia targeting anagen HFs characterized by sudden hair loss, leaving smooth alopecic areas; it can also occur as patchy alopecia of the scalp with multiple patches or a single patch that enlarges peripherally over time. Alopecia can also expand to include the entire scalp and the eyebrows (Alopecia totalis) or other hair-bearing areas of the body, like the arms, legs, groin, and underarms (Alopecia universalis) [17]. In 10 to 40% of patients, there is a family history of this disease. It has been related to HLA class II: HLA-DR4, HLA-DR5, and HLA-DQ3; in the Italian population, it is related to the allele DQB1*03(DQ7). It is an autoimmune disease caused by cytotoxic T lymphocytes (NKG2D+CD8+) that act against the hair follicle [13]. A dysfunction in the hair cycle results in dystrophic anagen follicles and an increased number of follicles in the telogen phase. A surge in interferon-gamma (IFN-γ), triggered by environmental or immunological insults, disrupts the immune privilege (IP) of the hair follicle (HF-IP), allowing immune cells to recognize and attack melanocyte-associated antigens within the follicle. This loss of immune privilege initiates an autoimmune response that targets follicular structures. IFN-γ also stimulates the follicular epithelium to produce interleukins IL-2 and IL-15, which in turn promote the activation and survival of CD8+ T cells. These CD8+ T cells further secrete IFN-γ, establishing a self-perpetuating IFN-γ/IL-15 inflammatory loop that sustains chronic inflammation and ongoing hair loss in AA. The JAK-STAT signaling pathway operates downstream of these cytokines and is instrumental in propagating this inflammatory cascade, with Janus kinases (JAKs) modulating genes involved in apoptosis and immune responses. The involvement of pigmented hair and regrowth of white hair suggests the presence of melanocyte-derived autoantigens. In AA, the anagen-phase follicles are infiltrated predominantly by CD4+ T cells, while a decrease in the number and function of regulatory CD8+ T cells is noted. Conversely, in sudden graying, an increase in cytotoxic CD8+ lymphocytes is observed, leading to selective loss of pigmented hairs. Genetic studies have shown associations between AA and polymorphisms in several interleukin genes, including IL-1β, which is overexpressed in affected scalp areas during early stages. Variants in the IL2RA gene, which encodes a key molecule on CD4+CD25+ regulatory T cells, also contribute to the pathogenesis of AA by impairing immune tolerance. Epigenetic changes influenced by environmental factors, such as diet, psychological stress, and acute illness, further modulate disease expression. AA has strong associations with other autoimmune conditions, including vitiligo, autoimmune thyroiditis, type 1 diabetes, rheumatoid arthritis, and collagen vascular diseases. Additionally, patients often exhibit increased rates of atopic dermatitis, asthma, allergic rhinitis, hearing loss, and deficiencies in iron and vitamin D [12,13].

Risk factors and comorbidities

Environmental factors and comorbidities can initiate or modify clinical manifestations of some types of alopecia. Research into nutritional, psychosocial, family, medical, and hair care management history has improved our understanding of the spectrum of this disease. It has opened our eyes to these factors, changing the basis of different treatments [17]. For instance, psychological stress, higher income, lack of physical activity, insufficient photoprotection, multiple marriages, smoking, hypertension, and diabetes mellitus have been related to FPHL. Of note, advanced-stage FPHL has been reported to be associated with aging, menopausal state, and hypertension, whereas acne vulgaris is related to the early stage of FPHL. Both hirsutism and acne vulgaris were more commonly seen in the Ludwig and Hamilton-Norwood-type than in those with Olsen-type hair loss, and hypertension was frequent in the Ludwig type [18,19]. On the contrary, alopecia can lead to psychiatric disorders; three articles reported the correlation between hair loss and psychiatric disorders without specifying the type of alopecia. Depression and other psychiatric disorders were more prevalent in alopecia patients than anxiety and acute stress [20]. Forneris and colleagues' study demonstrated a significant association between hair loss complaints and depressive symptoms, reporting that 38% of hair loss patients presented major depressive symptoms, such as anhedonia and sadness. Acute stress seems to be more associated with AA, while anxiety is more correlated with TE. In addition, AGA patients show more association with other psychiatric disorders such as social phobia and obsessive-compulsive disorder (OCD) [1,20]. Moreover, several studies have suggested that AA may be related to chronic immune-mediated inflammatory diseases, such as systemic lupus erythematosus, rheumatoid arthritis, psoriasis, and metabolic syndrome (through other mechanisms). Considering that these diseases have been proven to increase the risks of cardiovascular disease (CVD), AA and its autoimmune mechanism may not be confined to HFs. However, it may be a systemic inflammation that increases the risk of CVDs. O'Hagan et al. reaffirmed this theory in a retrospective cohort study in patients with AA, finding a significantly increased risk of acute myocardial infarction (AMI) over time during the 12-year follow-up period independent of cardiovascular risk profiles (CVRPs) [17].

Regardless of the specific etiology of alopecia, hair loss affects attractiveness and self-esteem [17]. Patients' dissatisfaction with their hair leads to overall body discontent and a concomitant decline in quality of life. The psychosocial impact seems more severe in females than males on average; however, males are also significantly affected. More than 25% of males with AGA find the hair loss to be extremely upsetting, and 65% express modest to moderate emotional distress [19]. It is relevant to know the established protocols for the diagnosis of each type of alopecia, the usefulness of imaging tools, innovations on the biochemical, cytological, or genetic field, and new diagnostic methods driven by the use of artificial intelligence that could allow more timely and accurate diagnoses to improve the course of each patient's disease [20].

Diagnostic approach

A thorough medical history and targeted questioning focused on endocrinopathies, autoimmune diseases, medication use, exposure to physical or chemical agents, hair care practices, excessive traction or scalp massages, combined with physical examination, are sufficient to determine the underlying etiology for hair loss. However, diagnosing scalp conditions requires a dermoscopic examination performed by a specialist [21]. Since more hair and scalp treatments are offered in non-specialist settings, new AI-based systems using imaging devices and an online deep-learning database for image classification have been proposed to diagnose scalp conditions. The prototype system (ScalpEye) comprises a portable scalp hair imaging microscope, a mobile application, a cloud-based artificial intelligence (AI) training server, and a cloud-based management platform. It was tested using different deep-learning modules and achieved 97-99% precision in detecting dandruff, folliculitis, hair loss, and oily hair [22].

Other machines and deep learning approaches have recently been used to facilitate alopecia areata diagnosis. The Severity of Alopecia Tool (SALT) captures the percentage of hair loss and density in AA patients. The scoring system is complex and difficult to generalize, affecting user reproducibility. To improve scoring reproducibility, an image database of 100 patients was annotated by a hair expert, and an algorithm was developed that could delineate regular and bald scalp areas and identify regions with low hair density. In a different study, a deep neural network (NN) was developed to determine the SALT score. NN is a computer system that learns and processes data using a structure inspired by the human brain. It is implemented in artificial intelligence (AI), machine learning (ML), and deep learning. Over 2000 images of 18 alopecia areata patients annotated by a dermatologist were used to train the network. To test the program, eight dermatologists assessed 400 images manually and with its assistance. The program-assisted approach improved accuracy and interrater reliability. Another machine learning method has also been proposed for the early diagnosis of alopecia areata. Healthy and affected individuals were classified based on hair and nail attributes. The NN classified healthy individuals and alopecia areata patients with 91% accuracy, testing its usefulness as a new diagnostic aid [23].

Nguyen et al. developed an artificial intelligence (AI) system for objectively quantifying AA in the adult population and assigning SALT scores by evaluating 188 images containing 47 sets of 4 views (left, right, top, back) for each subject; the images were taken during clinic visits using an iPad. Each image was independently scored by a human investigator (a veteran U.S. dermatologist) and by the AI system, with the score representing the percentage of hair loss in that image, then used intraclass correlation coefficient (ICC) to measure accuracy between scores by the doctor and the AI, based on a 95% confidence interval, ICC scores between 0.75 and 0.9 indicate good. Correlation and scores greater than 0.90 indicate excellent correlation. They obtained an ICC score of 0.97 for SALT scoring and 0.96, 0.97, 0.95, and 0.92 for left, right, top, and back view images. Most cases where the doctor and AI scores differed involved severe alopecia or patients with new, white, and short hair (the doctor scored 100% hair loss, but AI scored less; the error is about 10%). This data supports a potential role for AI techniques in assigning SALT scores [24].

A similar approach was employed to diagnose FPHL. There is no evidence-based quantitative prediction tool for therapeutic response in FPHL. However, artificial intelligence, particularly ML, has been utilized in medicine for predictive purposes and data-driven decision-making. ML algorithms learn from example data provided in the form of features. In one study, a machine learning algorithm analyzed crown images of 33 women with FPHL, and the algorithm determined that balding area width values significantly correlated with Savin scale hair loss grades. Another algorithm was developed to classify crown images obtained with a webcam based on the Hamilton-Norwood scale. The algorithm corrected for varying lighting conditions and accurately classified the degree of hair loss with 51%-95% accuracy, depending on the pattern. A different approach was used to detect male pattern baldness in facial images. Images were classified into three classes corresponding to types 1, 2-4, and 5-7 of the Hamilton-Norwood scale; 675 images were used. Three different NNs were tested, achieving an average accuracy of 82%-86% in image classification. The clinical implementation of deep learning-based approaches for hair loss scoring would enhance reproducibility and facilitate treatment follow-up [22].

Medical and pharmacological treatment

Hair loss treatment has progressed significantly, including pharmacological and noninvasive or minimally invasive techniques. These approaches aim to mitigate hair loss, stimulate regrowth, and improve patient satisfaction. Thanks to the combination of innovative technologies and well-established treatments, there are now many alternatives for treating different types of alopecia. This section details the mechanisms, effectiveness, challenges, and increasing evidence supporting these treatments.

Pharmacological treatment

Minoxidil

Minoxidil modulates the growth cycle and is pivotal in hair loss treatment. It shortens the telogen (resting) phase and extends the anagen (growth) phase, increasing hair follicle diameter and length. The mechanism of action involves the upregulation of vascular endothelial growth factor (VEGF), which enhances blood flow to the scalp and facilitates the delivery of oxygen and growth factors to hair follicles. Additionally, minoxidil activates potassium channels in hair follicles, prolonging the anagen phase and reducing the telogen phase [25]. Its immunomodulatory effects, such as modulating concanavalin A (Con A), may suppress T-cell activation, which is potentially beneficial in autoimmune alopecia [26].

Topical minoxidil is FDA-approved for both men and women and is available in 2% and 5% formulations. Besides AGA, topical minoxidil has been explored for other types of alopecia, including telogen effluvium (TE), alopecia areata (AA), traction alopecia (TA), frontal fibrosing alopecia (FFA), chemotherapy-induced alopecia, loose anagen hair syndrome, monilethrix, and before and after hair transplantation. However, these uses are off-label, and the efficacy can vary [27,28]. Clinical trials have consistently demonstrated the efficacy of 5% minoxidil in improving hair density, thickness, and regrowth compared to placebo and lower concentrations [29]. In men, a randomized clinical trial found that the 5% minoxidil formulation led to 45% greater regrowth than the 2% formulation after 48 weeks, with a quicker treatment response [30]. Similarly, in women, the 5% formulation was superior to placebo and demonstrated statistically significant benefits over the 2% formulation [31]. Paradoxical hair shedding may occur initially, as the treatment stimulates telogen follicles to re-enter the anagen phase. Recommended usage is 1 ml of the 5% solution or half a cupful of foam applied twice daily to dry scalp areas, with hair growth typically observed within 4 to 8 months and stabilization from 12 to 18 months. Discontinuation or irregular use often leads to rapid reversal of hair loss benefits, highlighting the importance of treatment adherence [25]. Common side effects include pruritus and irritation, which are more frequently observed with the 5% formulation [30,31]. These reactions are related to irritant or allergic contact dermatitis caused by propylene glycol (a solvent in the solution) rather than the minoxidil itself [32]. It has also been associated with a higher occurrence of hypertrichosis, particularly in women, characterized by unwanted hair growth in areas near the application site [33]. Both formulations are well-tolerated despite these effects, with no significant systemic adverse effects reported [30,31].

Although not FDA-approved for hair loss, oral minoxidil has been used off-label, showing promising results with tolerable side effects [34]. Low-dose oral minoxidil (LDOM), 1-5 mg/day, is being increasingly studied due to its potential benefits over topical formulations, notably improved patient compliance and ease of use [35-37]. Oral minoxidil has shown promising results for AGA, with response rates between 70% and 100% [38]. A randomized clinical trial comparing oral minoxidil to topical minoxidil in male AGA found no significant difference in efficacy, though oral treatment is associated with a higher incidence of hypertrichosis [39]. A recent meta-analysis found no difference in hair density or diameter between the formulations [40]. For AA, evidence is less robust but indicates potential benefits, with clinical improvement observed in 18-82.4% of patients [38]. However, the variability in response highlights the need for further research to standardize dosing and indications. Oral minoxidil has a favorable safety profile compared to other oral agents used for AA. Hypertrichosis, the most common side effect, is dose-dependent and occurs in 24% of patients [38,39]. Less common adverse effects include hypotension, lower limb edema, and heart rate changes, which are more likely at higher doses [37]. In females, AGA was exceptionally responsive to doses ranging from 0.25 mg to 1.25 mg, which were safe and effective. However, lower doses (0.25 mg) were less effective in males, with better results observed at 2.5 mg or 5 mg daily [37]. In summary, although oral minoxidil offers a viable alternative to topical formulations, additional randomized controlled trials are necessary to optimize dosing and clarify its long-term safety and efficacy profile.

5-Alpha Reductase Inhibitors

5-alpha reductase inhibitors are key pharmacological agents in managing AGA, primarily by targeting DHT, the hormone responsible for hair follicle miniaturization. These medications, particularly finasteride and dutasteride, have demonstrated significant efficacy in both slowing hair loss and promoting hair regrowth. However, their use requires careful consideration due to potential side effects and the necessity of long-term adherence.

Finasteride, an FDA-approved oral medication for male AGA, works by inhibiting the conversion of testosterone to DHT [34,41,42]. Administered at a daily dose of 1 mg, it has been shown to decelerate hair loss progression and facilitate regeneration, particularly in males [29]. Continuous use is necessary, as discontinuation leads to the loss of any gains in hair coverage. Despite its efficacy, its use remains controversial in males due to potential adverse effects such as reduced libido, erectile dysfunction, decreased ejaculatory volume, testicular pain, depression, and gynecomastia [41]. Although most side effects are mild and reversible upon discontinuation, a subset of patients may experience persistent sexual dysfunction [43]. The medication is contraindicated in premenopausal women and classified as pregnancy category X due to its teratogenic effects, precisely the potential harm to male fetuses [43]. For patients who wish to avoid systemic side effects, topical finasteride has emerged as a promising alternative. Though not FDA-approved for AGA, studies demonstrate its efficacy in reducing hair loss and increasing hair density [44]. Notably, a randomized, double-masked study reported that a combination of topical finasteride (0.25%) and 3% minoxidil significantly outperformed 3% minoxidil alone in improving hair growth over 24 weeks [44].

Dutasteride, another 5-alpha reductase inhibitor, inhibits both type I and type II isoforms of the enzyme, offering greater efficacy than finasteride, particularly in regrowth at the frontal scalp [34,41,42]. While not FDA-approved for AGA, it is increasingly used in refractory cases and has shown remarkable effectiveness in clinical trials. Its broader inhibition of DHT makes it a valuable option for patients who do not respond adequately to finasteride [45].

Hormonal Therapies

Spironolactone, a potassium-sparing diuretic with antiandrogen properties, is particularly effective for treating FPHL, especially in women with hyperandrogenism. Evidence suggests significant improvements in hair density and quality [46]. It reduces testosterone levels by inhibiting 17-alpha hydroxylase and 17, 20 lyase enzymes. Spironolactone combined with oral minoxidil has demonstrated superior efficacy to monotherapy [47]. Common adverse effects include postural hypotension, electrolyte imbalances, breast tenderness, and menstrual irregularities [48].

Flutamide and bicalutamide are nonsteroidal antiandrogens effective for FPHL, with notable differences in safety profiles. Flutamide, at a daily dose of 250 mg, is highly effective, particularly in hyperandrogenic women. A four-year study involving 101 women showed significant improvements in alopecia scores, especially after two years [49]. However, flutamide's use is limited by its potential to raise liver enzyme levels, particularly at higher doses. Bicalutamide is a safer alternative to flutamide, and it is better tolerated. In a study of 17 patients (50 mg daily or every other day for 24 weeks), 57% reported notable regrowth with minimal increases in liver enzymes [50].

Cyproterone acetate, a potent antiandrogen widely used in Europe (though not FDA-approved), effectively treats FPHL by inhibiting gonadotropin-releasing hormones and androgen receptors. A six-month study found that combining systemic cyproterone acetate with 5% topical minoxidil was effective and well-tolerated for female pattern hair loss, demonstrating improved hair regrowth compared to monotherapy [51]. Common side effects include weight gain, breast tenderness, and reduced libido, which should be carefully considered in treatment decisions [48].

Antiandrogen therapies, such as spironolactone, flutamide, bicalutamide, and cyproterone acetate, offer effective options for managing FPHL, particularly in hyperandrogenic women. While spironolactone and bicalutamide present safer profiles, flutamide and cyproterone acetate provide potent alternatives with specific considerations for side effects. Individualized treatment based on patient needs, hormonal status, and risk tolerance remains critical for optimizing outcomes. Novel topical antiandrogens, such as clascoterone, are also under investigation [52].

Janus Kinase Inhibitors (JAKi)

JAK inhibitors disrupt the inflammatory loop triggered by IL-15 and interferon-gamma signaling in AA, promoting hair regrowth. Tofacitinib achieved significant regrowth in 64% of severe AA patients and over 50% improvement in moderate cases [53]. Dose increases improved outcomes for non-responders. Ruxolitinib showed remarkable regrowth (92%) over three to six months in adult AA patients, although effects reversed upon discontinuation [54]. Baricitinib demonstrated significant regrowth in specific cases of chronic AA and alopecia universalis [55]. JAK inhibitors are linked to an increased risk of infections, including herpes zoster reactivation, thrombosis, and neutropenia [56].

Biologics

Biologics targeting the Th1/IFN-γ axis, implicated in the pathogenesis of alopecia areata (AA), offer potential treatment avenues. This axis involves Th1 cytokines, whose inhibitors, like adalimumab and etanercept, have been assessed in trials with mixed outcomes. Research also highlights the activation of IL-23, Th2, and Th17 cytokines, suggesting that targeting these pathways might benefit AA [57].

Prostaglandins

Prostaglandins play a key role in hair cycle regulation, with PGD2 inhibiting growth and PGE2/PGF2α promoting it [58]. AGA scalps exhibit increased PGD2 and reduced PGE2 levels [59].

Topical prostaglandins: Bimatoprost (a PGE2 analog) demonstrated increased hair diameter and count with 0.03% lotion in 12-16 weeks [60]. Latanoprost (a PGF2α analog) improved hair density in a 24-week trial with male patients [61]. Cetirizine reduced PGD2 levels and significantly enhanced hair density in a six-month pilot study [62]. Further research with larger samples and extended durations is needed to confirm efficacy.

Oral prostaglandins: Setipiprant, a PGD2 receptor antagonist, showed variable but non-significant results in improving hair count in a phase 2a trial [63]. While it demonstrated some potential, inconsistencies suggest further studies with more uniform participant groups to establish efficacy. Table 4 summarizes the pharmacologic treatment of hair loss.

**Table 4:** Pharmacologic treatment for alopecia AGA: androgenetic alopecia; TE: telogen effluvium; AA: alopecia areata; FFA: frontal fibrosing alopecia; TA: traction alopecia; FPHL: female pattern hair loss; VEGF: vascular endothelial growth factor; DHT: dihydrotestosterone Table created by the authors using data from: [48,56]

Treatment	Mechanism	Indications	Adverse effects
Topical			
Minoxidil	Shortens telogen phase, prolongs anagen phase, enhances blood flow via VEGF upregulation	AGA, TE, AA, FFA, TA, Chemotherapy-induced alopecia, Loose anagen hair syndrome, Monilethrix	Pruritus, irritation, hypertrichosis, initial shedding
Finasteride	Inhibits 5-alpha reductase, blocks formation of DHT	AGA	Irritation, erythema, contact dermatitis
Clascoterone	Androgen receptor inhibitor	AGA (experimental)	Weight gain, breast tenderness, decreased libido
Latanoprost and bimatoprost	Prostaglandin analogs; prolong the anagen (growth) phase	AGA (experimental)	Irritation, hypertrichosis
Systemic			
Low-dose Oral Minoxidil	Shortens telogen phase, prolongs anagen phase, enhances blood flow via VEGF upregulation	AGA, TE, AA, FFA, TA, Chemotherapy-induced alopecia, Loose anagen hair syndrome, Monilethrix	Hypertrichosis, hypotension, lower limb edema, heart rate changes
Finasteride	Type II 5-alpha-reductase inhibitor; blocks the formation of DHT	AGA	Reduced libido, erectile dysfunction, depression, gynecomastia
Dutasteride	Type I and II 5-alpha-reductase inhibitors; blocks the formation of DHT	AGA	Reduced libido, erectile dysfunction, depression, gynecomastia
Spironolactone	Antiandrogen; decreases testosterone	AGA (FPHL)	Postural hypotension, electrolyte imbalances, breast tenderness, menstrual irregularities
Flutamide and bicalutamide	Antiandrogen; blocks testosterone	AGA (FPHL)	Elevated liver enzymes, headache, breast tenderness
Cyproterone acetate	Antiandrogen; blocks gonadotropin-releasing hormone and androgen receptors	AGA (FPHL)	Weight gain, breast tenderness, reduced libido
JAK inhibitors	Inhibit the JAK-STAT pathway, reduce inflammatory cytokine signaling	AA	Increased infection risk, thrombosis, neutropenia
Biologics	Target specific cytokine pathways like IL-4/IL-13 or IL-12/IL-23	AA	Injection site reactions, conjunctivitis, respiratory infections
Injectables			
Dutasteride	Type I and II 5-alpha-reductase inhibitor; blocks the formation of DHT in the hair follicle	AGA	Scalp pain

Noninvasive and minimally invasive treatment of hair loss

Noninvasive and minimally invasive treatments have emerged as viable alternatives or adjuncts to pharmacological therapies. These approaches stimulate natural hair growth through innovative technologies and regenerative methods.

Low-Level Laser Therapy (LLLT)

LLLT utilizes red or near-infrared light to stimulate cellular activity, increase blood flow, and enhance follicular regeneration. This noninvasive treatment has been FDA-cleared for androgenetic alopecia. It involves using laser devices that emit light at specific wavelengths to stimulate hair growth. In clinical trials, LLLT has shown efficacy in increasing hair density and thickness [29,64]. Meta-analyses rank LLLT as one of the most effective noninvasive treatments for hair loss, particularly in combination with pharmacological agents [29]. However, inconsistent treatment protocols and device efficacy variability remain challenging [65].

Platelet-Rich Plasma (PRP) Therapy

PRP, which involves injecting autologous platelet concentrates into the scalp, delivers growth factors that stimulate follicular proliferation and angiogenesis. PRP has shown significant efficacy in increasing hair density and thickness. Combination therapies, such as PRP with microneedling or fractional laser, enhance these effects [66]. While some studies have shown positive results, the evidence is heterogeneous, and standardized protocols are lacking [29,34,67].

Microneedling

Microneedling creates micro-injuries that trigger wound healing, enhance growth factor release, and absorb hair growth-promoting agents like minoxidil and PRP. One notable clinical study involving 100 male AGA patients assessed the efficacy of microneedling combined with 5% topical minoxidil applied twice daily over 12 weeks. This randomized, evaluator-blinded controlled trial demonstrated that patients in the microneedling group experienced a significant increase in hair count, with an average of 91.4 hairs per cm², compared to 22.2 hairs per cm² in the minoxidil-only group [68]. Synergistic benefits are also reported when microneedling is paired with PRP or LLLT [69]. It is generally safe but requires further research to define optimal protocols.

Mesotherapy

Mesotherapy delivers therapeutic agents directly into the mesoderm via microinjections, targeting hair follicles for AGA treatment. A significant advantage of mesotherapy is that it allows therapeutic agents to be administered directly into the skin, bypassing the barriers typically encountered by topical treatments and thereby providing a targeted approach to therapy. This is thought to increase the bioavailability of the agent due to its prolonged presence and direct contact with the targeted area [70]. In the study, 23 males in the mesotherapy group observed up to a 25% improvement in hair loss and growth, while two males experienced hair growth improvements ranging from 25% to 50%. On the other hand, all males in the minoxidil group reported improvements in hair loss and growth of up to 25%. Additionally, mesotherapy proved more practical for a few males with AGA, affecting the vertex area than in the frontal region, with assessments using the standard seven-point tool indicating moderate increases and patient self-assessment scores showing improvements between 26% and 50% [71]. While effective, mesotherapy may cause localized side effects such as erythema, pain, and swelling [70].

Surgical interventions

Surgical hair restoration has become an efficient and transformative solution for managing hair loss, especially in androgenetic alopecia and other severe forms of alopecia [71]. Over the past decades, innovations in surgical procedures have transformed outcomes, ensuring natural aesthetics, reducing invasiveness, and improving patient satisfaction. The two main methods are follicular unit transplantation (FUT) and follicular unit extraction (FUE). These techniques concentrate on transplanting follicular units, naturally in groups of one to four hair follicles, from a donor site (usually the occipital part of the scalp) to the recipient site [72,73].

Follicular Unit Transplantation (FUT)

FUT, also known as the strip method, involves removing a strip of scalp from the donor area, usually the back or sides of the head. The strip is then analyzed under a microscope to isolate individual follicular units, which are subsequently transplanted to the recipient location. This technique is especially beneficial for patients requiring extensive hair restoration, as it facilitates transplanting a substantial quantity of grafts in one session [74]. Although FUT offers high graft yield and predictable results, it results in a linear scar at the donor site, which may be a cosmetic issue for specific individuals [75,76].

Follicular Unit Extraction (FUE)

FUE is a minimally invasive hair restoration technique that directly extracts individual follicular units from the donor site utilizing a punch tool. In contrast to FUT, FUE does not leave a linear scar; instead, it results in small, circular scars that are generally less noticeable [74]. FUE is very attractive to people who wish to wear short hairstyles or a scar-free look. FUE provides faster recovery times and reduced postoperative discomfort compared to FUT [74]. The technique has been enhanced by robotic systems, which improve precision graft harvesting and implantation, reducing trauma to the donor site [77].

However, FUE is more time-consuming and may yield fewer grafts per session than FUT, which can be a limitation for patients needing extensive coverage. Additionally, if not performed carefully, there is a risk of overharvesting, resulting in a "moth-eaten" appearance [75,76]. Despite this, FUE continues to gain popularity among patients and surgeons due to its aesthetic advantages and versatility.

AI-Assisted FUE: ARTAS

Artificial intelligence (AI)-assisted technology is also used for hair loss treatment. The ARTAS system, the only FDA-approved robotic hair restoration system, enables image-guided follicular unit excision (FUE). This system employs patented AI technology to detect follicular units' location, angle, and direction. It then randomly guides a robotic arm to excise the FUs, minimizing scarring. To ensure accurate FU detection and excision, hair has to be trimmed. The latest system version allows operators to program a digital recipient site plan, enabling automated FU transplantation [22], making your hair restoration procedure virtually undetectable. Patients can expect clinically proven results without the side effects and prolonged recovery time familiar with older procedures. A study compared the advantages and disadvantages of ARTAS technology with traditional FUE. Thirteen Chinese male patients with Norwood-Hamilton II-IV AGA, aged 25-35 years, participated in a single-center, prospective, randomized, single-blinded, controlled study. Each patient's donor site was randomly divided into left and right regions, with ARTAS on one side and FUE on the other. The yield, transaction, and discard rates of hair FUs from both sides were also investigated. The results showed that the total yield rate on the ARTAS side was lower than on the FUE side (82.05% vs. 90.03%, p > 0.05). The total discard rate on the ARTAS side was higher than on the FUE side (10.71% vs. 5.46%, p < 0.05). However, the total transection rate on the ARTAS side was lower than on the FUE side (13.17% vs. 13.96%, p > 0.05). No significant difference was found in patient satisfaction, and no side effects or complications were detected during or after all surgeries [78].

Advances in deep learning technology may enable the development of systems that predict how a patient's hair loss pattern will evolve. This could allow accurate delineation of donor and recipient areas.

Artistry in hair restoration surgery

Hair restoration surgery combines precision with aesthetic artistry to achieve natural and imperceptible results. Essential components of artistry include hairline design, density distribution, and the placement of the hair graft with natural hair patterns.

Principles of Hairline Design

A natural hairline functions as the visual frame of the face. The successful design considers age, ethnicity, and facial structure, incorporating micro-irregularities and single follicular units at the frontal margin to ensure a smooth transition from the forehead to denser hair regions. Strategic placement of multi-follicular grafts guarantees uniform density while preventing unnatural outcomes [79].

Precision in Graft Placement

Compelling artistry emphasizes the creation of a realistic transition zone by using fine hairs to replicate the natural progression of density. Each graft is carefully oriented to align with the surrounding hair direction and angulation, ensuring a cohesive appearance [80].

Stem cell (SC)-based therapies for alopecia

Novel SC-derived therapies have emerged for AGA, AA, and other forms of alopecia. These therapies utilize adult tissue-specific derived SCs, such as mesenchymal stromal cells (MSCs), SC-derived chemokines or growth factors (secretome), and biomaterials with regenerative properties.

SC-based therapies for alopecia can be categorized as autologous or allogeneic. They can involve the use of isolated SCs with or without manipulation or the utilization of SC-derived secretome or extracellular matrix (ECM). Research has shown that the remodeling of the adult dermis in response to sustained epidermal Wnt/ß-catenin activation is mediated by secreted signals with differential effects on different fibroblast subpopulations, playing important roles in regenerating or restoring tissues such as hair [69,81]. Adult tissue sources of MSCs with multipotent regenerative potential include adipose tissue, bone marrow, hair follicles, and perinatal sources such as the placenta and umbilical cord blood [82].

Conditioned medium (CM) enriched with these substances has been shown to promote hair growth. Freshly derived primary multipotent MSCs, part of the stromal vascular fraction (SVF), can enhance the dermal papilla's capacity to grow or regenerate hair. Perez-Meza et al. have demonstrated the safety and efficacy of fat graft procedures in patients with inherited alopecia, with significant improvements in hair density observed [83]. Recently, a randomized controlled trial (RCT) investigated the synergistic effects of stem cell therapy combined with FUE on scarred scalp tissue. Histologic results showed that stem cell therapy significantly improved hair density and graft survival rates compared to FUE alone, with enhanced neovascularization and reduced fibrosis observed in the stem cell-treated group [84].

BM-MSCs have also been explored. BM harvesting is a highly invasive and painful procedure, implying general anesthesia and many days of hospital care. An animal model study used CM from supernatants of cultured BM mesenchymal cells overexpressing Wnt1a (Wnt-CM). It investigated its effect on hair regeneration in mice and its wound-healing properties. Their results revealed that Wnt-CM accelerates the biological progression of hair follicles from the telogen to the anagen phase and increases the number of hairs. In vitro experiments demonstrated that Wnt-CM can restore and maintain the hair induction ability of intermediate follicles and DP cells damaged by DHT [85].

On the other hand, in 2018, Elmaadawi et al. performed a human clinical trial studying the safety and efficacy of autologous bone marrow-derived mononuclear cells (BMMSC) in comparison to follicular hair stem cells (FHSC) in 20 patients with AA and 20 patients with AGA [86]. In this study, each patient received one intradermal dose of either BMMCs or FHSCs, and the impact was evaluated via immunostaining and digital dermoscopy after six months. No studied patients experienced any side effects, and all subjects displayed significantly improved hair growth with no significant difference between the groups. This study highlights the role of hematopoietic stem cells in hair regeneration, immunomodulatory functions, homing to inflammation sites, anti-inflammatory effects, multipotency, and secretion of VEGF, which controls hair growth and follicle size through angiogenesis [86]. Unfortunately, no other human study has been reported, limiting their valuable contribution.

## Conclusions

In today’s rapidly advancing technological era, and with the introduction and use of AI, the precision of an adequate diagnosis is continuously being studied and improved. Emerging non-invasive and minimally invasive treatments for hair loss have become popular in recent years. These include LLLT, PRP therapy, microneedling, and mesotherapy, among which only LLLT has FDA approval for hair loss, specifically for androgenetic alopecia. Studies show promising results from all the treatments mentioned, especially in combination with some pharmacological agents. However, the obstacles to overcome in all these therapies include consistent treatment protocols, sufficient clinical studies, and standardized guidelines. This contributes to physician preference and individualized treatment, resulting in inconsistent protocols through which individual experience remains the deciding factor in including these treatments.

Hair transplantation remains the most efficient and transformative solution among surgical treatments for hair loss. Two traditional methods of hair harvesting are the most used: FUT and FUE. FUT is in cases needing extensive hair restoration, and FUE is in patients who desire to preserve aesthetic results without fear. Hair restoration surgeons increasingly prefer FUE due to its aesthetic advantages and reduced discomfort after surgery. Among the technological advances in hair restoration surgery lies the ARTAS system, which uses AI and robotic assistance to excise follicular units independently. This is the only system of its kind with FDA clearance and sets the bar for future development in surgical assistance devices. Results through any of these surgeries remain dependent on the surgeon's artistry, which involves an adequate hairline design and graft placement.
